# Are online workers amenable to digital interventions? Results of a fully-remote nationwide trial of behavioral activation in 804 depressed adults in the United States

**DOI:** 10.1192/j.eurpsy.2025.306

**Published:** 2025-08-26

**Authors:** L. Lorenzo-Luaces, A. Peipert

**Affiliations:** 1 Department of Psychological and Brain Sciences, Indiana University, Bloomington, United States

## Abstract

**Introduction:**

Online workers are individuals who participate in crowdsourced work. They experience a higher rate of internalizing symptoms than the general population, a phenomenon dubbed the “Turker blues.” Three large trials of online single-session interventions (SSIs) failed to find statistical or clinically significant treatment effects in this population. Because these trials tested SSIs, it is unclear if online workers are unresponsive to SSIs in general or online interventions specifically. Moreover, participants in these studies were not selected based on their desire to learn skills online, raising the possibility that intervention effects would be present in treatment-seeking individuals.

**Objectives:**

We conducted a nationwide, fully remote two-arm randomized (1:1) controlled trial to test the efficacy of a 4-week self-guided online behavioral activation treatment for depression in online workers in the United States (N=804). The intervention was designed as an extension of the Common Element Toolbox (COMET), an SSI previously found ineffective in online workers.

**Methods:**

804 online workers were randomized to COMET-BA or a waitlist control (WLC). Self-report measures of depression, anxiety, subjective well-being, behavioral activation, psychosocial function, and emotion regulation were collected weekly for 4 weeks, 1-week post-intervention, then at a 1-month follow-up.

**Results:**

There was a significant time-by-treatment interaction during the intervention phase on the study, suggesting those in the COMET-BA arm improved significantly more than those in the WLC, with a small-medium effect on depression symptoms (SMD=-0.32; 95% CI: -0.47, -0.17). All but two outcomes, suppression and functioning, demonstrated significant improvement. Improvements were maintained during the 1-month follow-up period but did not grow during this period. Attrition was comparable between the treatments and the results were sensitive to missing data imputation.

**Conclusions:**

COMET-BA may be effectively delivered as an unguided online intervention for depression in online workers. Crucially, intervention effects were evident after the second iBA session, suggesting that SSIs may not be effective in adult online workers.

**Disclosure of Interest:**

L. Lorenzo-Luaces Consultant of: I have consulted with Syra Health, Inc. who had no involvement in the current research, A. Peipert: None Declared

